# Isolation and characterization of microsatellite loci from *Pterocarya stenoptera* (Juglandaceae)

**DOI:** 10.1002/aps3.1205

**Published:** 2018-12-05

**Authors:** Peng‐Fei Wang, Yong Li, Zhi‐Hao Qian, Jia‐Xin Li, Xue‐Jun Ge

**Affiliations:** ^1^ Innovation Platform of Molecular Biology College of Forestry Henan Agricultural University Zhengzhou 450002 People's Republic of China; ^2^ South China Botanical Garden Chinese Academy of Sciences Guangzhou 510650 People's Republic of China

**Keywords:** Illumina sequencing, Juglandaceae, microsatellite marker, *Pterocarya stenoptera*

## Abstract

**Premise of the Study:**

Microsatellite markers of *Pterocarya stenoptera* (Juglandaceae) were developed for future studies on the population genetic diversity and spatial genetic structure of the species.

**Methods and Results:**

Based on Illumina sequencing of the transcriptome of *P. stenoptera*, a total of 2452 microsatellites were identified from 83,674 assembled unigenes. One hundred microsatellites were randomly selected to design amplification primer pairs. Of these, 15 were successfully amplified and displayed polymorphism. For these markers, the number of alleles per locus and population ranged from one to six. The levels of observed and expected heterozygosity varied from 0.000 to 1.000 and 0.000 to 0.718, respectively. Furthermore, all of the 15 loci were successfully cross‐amplified in another congeneric species (*P. hupehensis*) and were demonstrated to be polymorphic.

**Conclusions:**

The microsatellite loci described here can be used for future population genetic and landscape genetic studies on *P. stenoptera*.

*Pterocarya stenoptera* C. DC. (Juglandaceae) is a deciduous broad‐leaved tree that is native to China and the Korean Peninsula. The species is cultivated as an urban landscaping tree because of its large crown and graceful drooping racemes. Previous studies on this plant have focused on its cultivation physiology (Yang et al., 2013; Xu et al., 2015; Yang and Li, 2016). To date, there have been no reports of the genetic diversity and population structure of *P. stenoptera*. Fan et al. (2013) developed 22 microsatellite loci for *P. stenoptera* using primer pairs from *Cyclocarya paliurus* (Batal.) Iljinsk. This number of microsatellite loci is insufficient for landscape genetic studies, which require the molecular markers to be distributed within the genome with high density (Hall and Beissinger, 2014). As an important urban landscaping tree, it is necessary to develop a large number of reliable molecular markers for the assessment of wild germplasm resources and the development of molecular‐assisted breeding.

Next‐generation sequencing makes it possible to rapidly isolate a large number of microsatellite markers (Kumar et al., 2014). Here, we report the isolation of transcript‐based microsatellite markers for *P. stenoptera* using Illumina shotgun sequencing. The transcript‐based microsatellite markers are more suited to locating adaptive loci in landscape genetic studies. Although loci identified through neutral markers can be linked to adaptive genes, microsatellites found within genic regions can provide a clearer link to a causal gene.

## METHODS AND RESULTS

A total of 60 individuals of *P. stenoptera* were sampled from three wild populations in Henan and Shandong provinces (Nanzhao, Henan Province [HNNZ]; Jigong Mountain, Henan Province [HNJG]; Meng Mountain, Shandong Province [SDMM]; Appendix 1). Twenty individuals of *P. hupehensis* Skan were sampled from one natural population in Henan Province (Yaoshan, Henan Province [HNYS]; Appendix 1). Vouchers were deposited at the herbarium of the College of Forestry, Henan Agricultural University, Zhengzhou, China (Appendix 1).

Total RNA was extracted from fresh leaves of one cultivated *P. stenoptera* individual in Henan Agricultural University using a Quick RNA Isolation Kit (BioTeke, Beijing, China) according to the standard protocol of the manufacturer. RNA concentration was estimated using the UV‐Vis spectrophotometer (catalog no. ND5000; BioTeke). The construction of a cDNA library and transcriptome sequencing were completed by the Biomarker Biotechnology Corporation (Beijing, China). Sequencing was performed on an Illumina HiSeq 2500 system (Illumina, San Diego, California, USA). We obtained a total of 20,410,837 paired‐end reads of 150 bp (6.10 Gbp; GenBank Sequence Read Archive accession no. SRP154982). The raw reads were subsequently cleaned by removing adapter sequences using Trimmomatic version 0.35 (Bolger et al., 2014) and then assembled using Trinity version 2.6.6 with the default parameters (Grabherr et al., 2011), resulting in a total of 83,674 unigenes. Microsatellites from the unigenes were identified using MISA (Thiel et al., 2003). The filtering conditions of microsatellites are as described in Shi et al. (2018). Parameters were designed for dinucleotides with a minimum of six repeats, and for tri‐, tetra‐, penta‐, and hexanucleotides with a minimum of five repeats. Of these unigenes, 2452 containing microsatellites were selected and deposited in GenBank (MH676104–MH678555). The first 100 microsatellites were selected to design amplification primer pairs using Primer3 (Rozen and Skaletsky, 1999). The primer design parameters were set using the default values except product size = 100–400 bp. These primers were then tested in two steps. First, the 100 primer pairs were tested on four randomly selected *P. stenoptera* individuals. PCR was conducted in a 20‐μL PCR reaction mixture consisting of 20 ng of genomic DNA, 10 mM of PCR buffer, 0.2 mM of each dNTP, 0.2 μM of each primer, and 1 unit of *Taq* polymerase (Tiangen, Beijing, China). In a Mastercycler Nexus thermocycler (Eppendorf, Hamburg, German), PCR was started with an initial denaturation at 94°C for 5 min; followed by 35 cycles at 94°C for 40 s, locus‐specific annealing temperature for 40 s, and 72°C for 40 s; a final extension at 72°C for 5 min; and hold at 4°C. PCR products were visualized on 1.5% agarose gel with a DL2000 DNA ladder (TaKaRa Biotechnology Co., Dalian, Liaoning, China). Of the 100 primer pairs, 43 were unsuccessful or had off‐target amplification and 57 were retained for second step validation. The remaining 57 primer pairs were then tested on 12 *P. stenoptera* individuals each from populations HNNZ and SDMM. PCR reaction conditions were the same as above. PCR products were separated by gel electrophoresis on 8% native polyacrylamide gel and visualized by silver staining. The band size was reported using a 50‐bp DNA ladder (TaKaRa Biotechnology Co.). Of the 57 primer pairs, 42 were not considered further due to single or excessive bands, and the remaining 15 primer pairs exhibited polymorphism (Table 1). To further assess the polymorphism of these microsatellites, genotyping was performed on all sampled individuals of *P. stenoptera*. The forward primers were 5′ labeled with FAM, HEX, or TAMRA. All reaction conditions were the same as above. PCR products were analyzed on an ABI 3730 DNA Analyzer (Applied Biosystems, Foster City, California, USA) at BGI (Beijing, China). PCR product size was measured according to GeneScan 500 ROX Size Standard (Applied Biosystems). The peaks of the microsatellite loci were read using GeneMarker 2.2.0 (SoftGenetics, State College, Pennsylvania, USA). Observed heterozygosity (*H*_o_), expected heterozygosity (*H*_e_), and Hardy–Weinberg equilibrium (HWE) were obtained using GENEPOP 4.2 (Rousset, 2008). Number of alleles (*A*) and inbreeding coefficient (*F*_IS_) were estimated using FSTAT 2.9.3.2 (Goudet, 1995).

**Table 1:** Primer sequences and characterization for 15 polymorphic microsatellite loci isolated from *Pterocarya stenoptera*

Locus	Primer sequences (5′–3′)	Repeat motif	*T*_a_ (°C)	Allele size range (bp)	Fluorescent label	GenBank accession no.	BLAST top hit		
							Putative function	GenBank accession no.	*E*‐value
Ps2	F: GTTACACTCAGTCCTCCGGC	(AC)_6_	48	260–262	FAM	MH676105	Methylesterase 17‐like [*Fragaria vesca* subsp. *vesca*]	XP_004307746.1	2.605E‐98
	R: TCCCGATTCCCTTCTTTCTT								
Ps13	F: ACGGCGTAGATTTCATCGTC	(CCT)_6_	52	184–195	TAMRA	MH676116	Hypothetical protein PHAVU_008G280600g [*Phaseolus vulgaris*]	XP_007142439.1	0.000
	R: TTTTTGGTTCATTGCTGTGC								
Ps23	F: GACGGCACATATTTTCAATTC	(GA)_9_	54	236–246	HEX	MH676126	Hypothetical protein PRUPE_ppa007664mg [*Prunus persica*]	XP_007222111.1	0.000
	R: GATTGAGTTGCGAGGAAAGC								
Ps27	F: AGCTTCTCGGAGAGTTGCAG	(TC)_9_	48	217–236	TAMRA	MH676130	U‐box domain‐containing protein 4 [*Prunus mume*]	XP_008229780.1	0.000
	R: AACCCCAAAGGATATAACGGA								
Ps28	F: AGCGAGTCCTGGTAAGACGA	(AG)_6_	48	275–281	FAM	MH676131	RING‐H2 finger protein ATL47 [*Eucalyptus grandis*]	XP_010025372.1	3.775E‐136
	R: CCGCCCTTAATTCACAAAAA								
Ps29	F: GCTCTTCCTCGGAGCTCTTT	(ACC)_5_	58	114–150	TAMRA	MH676132	rho GTPase‐activating protein 2 [*Vitis vinifera*]	XP_002270566.1	0.000
	R: GAGACTCCACACGGTTGGTT								
Ps31	F: ACTTGCTGGAGTAAGCTCGC	(AT)_6_	50	227–236	HEX	MH676134	Hypothetical protein L484_002942 [*Morus notabilis*]	XP_007206294.1	1.147E‐60
	R: TTCACCGACTTGTAAAGGGG								
Ps53	F: AAAAGCACCTTGCCATTTTG	(GT)_6_	60	114–149	TAMRA	MH676156	Protein strawberry notch [*Vitis vinifera*]	XP_003634816.1	0.000
	R: CCAAATCCCAAAAACAAACC								
Ps59	F: TGGCTGAGGTGTCTCTTCCT	(CAT)_7_CC(TCA)_5_	62	182–188	TAMRA	MH676162	Prostatic spermine‐binding protein‐like [*Prunus mume*]	XP_008219536.1	1.097E‐09
	R: ATGATGGTGGTGAGGGTGAT								
Ps65	F: TGCTCTTGAAATCGATACGC	(AT)_6_	56	260–268	FAM	MH676168	No hit	—	—
	R: ACGAGGCCAGATTATTGCAG								
Ps69	F: CTCCTCCTCCAAGCCCTAGT	(CTC)_5_	56	236–253	HEX	MH676172	Hypothetical protein Csa_5G601540 [*Cucumis sativus*]	KGN51804.1	0.000
	R: GGAGACGATTCAGCATTGGT								
Ps70	F: CATGTCCCAGCTCCGATACT	(AAT)_6_	52	209–213	HEX	MH676173	No hit	—	—
	R: AAACAGCTGTCCCCGTATTG								
Ps75	F: ACCGTGAACGAAGCTCAGTC	(GCG)_6_	60	243–284	FAM	MH676178	Unnamed protein product [*Vitis vinifera*]	CBI32594.3	8.241E‐45
	R: CTCTAGCTCCTCCAGTCCCC								
Ps82	F: GGACGATGAGGACGAAGAAG	(GAT)_5_	48	204–231	HEX	MH676185	Pentatricopeptide repeat‐containing protein At1g09900 [*Vitis vinifera*]	XP_002272135.2	0.000
	R: CTCAAGCTTTGGGTTCCAAG								
Ps83	F: TCACGGTGTGTAGAACCGAC	(AG)_6_	62	127–131	TAMRA	MH676186	Conserved hypothetical protein [*Ricinus communis*]	XP_002510114.1	1.281E‐30
	R: GACTAACAACAGGCCACGGT								

Note

*A* = number of alleles; *T*_a_ = annealing temperature.

In *P. stenoptera*,* A*,* H*_o_, *H*_e_, and *F*_IS_ ranged from one to six, 0.000–1.000, 0.000–0.718, and −0.583–0.620, respectively (Table 2). Of the 15 polymorphic microsatellite loci, three, four, and three loci showed significant deviations from HWE in populations HNNZ, SDMM, and HNJG, respectively (Table 2). The cross‐amplification of these markers was tested in 20 individuals of *P. hupehensis*. All markers were successfully amplified, and *A* per locus ranged from one to nine (Table 3).

**Table 2:** Genetic diversity parameters for 15 polymorphic microsatellite loci in three populations of *Pterocarya stenoptera*.^a^

Locus	HNNZ population (*N* = 20)				SDMM population (*N* = 20)				HNJG population (*N* = 20)			
	*A*	*H*_e_	*H*_o_	*F*_IS_	*A*	*H*_e_	*H*_o_	*F*_IS_	*A*	*H*_e_	*H*_o_	*F*_IS_
Ps2	2	0.050	0.050	0.000	2	0.053	0.053	0.000	2	0.056	0.056	0.000
Ps13	2	0.482	0.500	−0.016	4	0.405	0.474	−0.174	3	0.398	0.333	0.167
Ps23	6	0.441	0.421	0.046	4	0.468	0.250*	0.472	4	0.486	0.611	−0.268
Ps27	6	0.653	0.700	−0.075	3	0.417	0.389	0.070	2	0.143	0.143	0.000
Ps28	4	0.483	0.300	0.385	3	0.465	0.421	0.097	5	0.718	0.882*	−0.237
Ps29	4	0.388	0.150*	0.620	5	0.579	0.400*	0.315	3	0.300	0.222	0.265
Ps31	4	0.645	1.000*	−0.573	4	0.562	0.737	−0.323	5	0.592	0.778	−0.326
Ps53	2	0.235	0.158	0.333	5	0.579	0.350*	0.402	6	0.450	0.316*	0.303
Ps59	3	0.199	0.211	−0.059	1	0.000	0.000	—	2	0.056	0.056	0.000
Ps65	5	0.658	0.800*	−0.223	3	0.642	0.750	−0.173	4	0.684	0.632	0.079
Ps69	3	0.304	0.300	0.013	3	0.152	0.158	−0.038	3	0.383	0.412	−0.077
Ps70	2	0.475	0.611	−0.299	2	0.422	0.368	0.131	2	0.513	0.800*	−0.583
Ps75	4	0.633	0.667	−0.054	3	0.411	0.421	−0.025	6	0.504	0.600	−0.197
Ps82	1	0.000	0.000	—	4	0.191	0.100*	0.483	1	0.000	0.000	—
Ps83	2	0.286	0.222	0.227	3	0.465	0.474	−0.019	2	0.059	0.059	0.000

Note

*A* = number of alleles; *F*_IS_ = inbreeding coefficient; *H*_e_ = expected heterozygosity; *H*_o_ = observed heterozygosity; *N* = number of individuals tested.

a Voucher and locality information are provided in Appendix 1.

*Significant deviation from Hardy–Weinberg equilibrium (*P* < 0.05).

**Table 3:** Cross‐amplification of 15 polymorphic microsatellites developed for *Pterocarya stenoptera* in *P. hupehensis*.^a^

Locus	Allele size (bp)	*A*	*H*_e_	*H*_o_	*F*_IS_
Ps2	260	1	0.000	0.000	—
Ps13	175–187	4	0.431	0.316*	0.273
Ps23	240–263	9	0.874	0.800	0.087
Ps27	217–229	6	0.359	0.350	0.026
Ps28	277–287	4	0.476	0.450	0.055
Ps29	114–150	3	0.278	0.056*	0.738
Ps31	213–234	5	0.628	0.800	−0.283
Ps53	114–139	7	0.740	0.556	0.254
Ps59	176–197	5	0.496	0.474*	0.047
Ps65	262–274	6	0.804	0.588	0.274
Ps69	247–250	2	0.328	0.300	0.088
Ps70	209–216	3	0.494	0.278	0.444
Ps75	249–270	5	0.675	0.556	0.181
Ps82	201–214	3	0.160	0.056*	0.660
Ps83	127–129	2	0.322	0.278	0.141

Note

*A* = number of alleles; *F*_IS_ = inbreeding coefficient; *H*_e_ = expected heterozygosity; *H*_o_ = observed heterozygosity; *N* = number of individuals tested.

a Voucher and locality information are provided in Appendix 1.

*Significant deviation from Hardy–Weinberg equilibrium (*P* < 0.05).

## CONCLUSIONS

In this study, 2452 microsatellites were discovered in the *P. stenoptera* transcriptome. One hundred primer pairs were randomly selected to verify the efficiency of amplification. Of these tested primer pairs, 15 displayed polymorphism. This is the first set of microsatellite markers developed specifically for *P. stenoptera*; it will be helpful for future population genetic and landscape genetic studies of the species. All markers were successfully amplified in the related species *P. hupehensis*, suggesting that they may also be used to study other related species in *Pterocarya*.

## DATA ACCESSIBILITY

Raw reads were submitted to the National Center for Biotechnology Information (NCBI) Sequence Read Archive (accession no. SRP154982). Unigenes containing microsatellites were deposited in GenBank (MH676104–MH678555). Sequence information for the developed primers has been deposited to NCBI; GenBank accession numbers are provided in Table 1.

## APPENDIX 1 Voucher and locality information for *Pterocarya* species used in this study.^a^

**Table:**

Species	Population code	Voucher no.	Collection locality	Geographic coordinates	*N*
*Pterocarya stenoptera* C. DC.	HNNZ	LiPS2017001	Nanzhao, Henan, China	33°35′24″N, 112°10′48″E	20
	HNJG	LiPS2017002	Jigong Mountain, Henan, China	31°48′36″N, 114°04′48″E	20
	SDMM	LiPS2017003	Meng Mountain, Shandong, China	35°33′36″N, 117°57′36″E	20
*Pterocarya hupehensis* Skan	HNYS	LiPH2018001	Yaoshan, Henan, China	33°42′22″N, 112°20′00″E	20
